# Clinical Presentation and Treatment Response in Women With Acetylcholine‐Confirmed Coronary Spasm: A Single‐Center Observational Study

**DOI:** 10.1002/clc.70424

**Published:** 2026-07-21

**Authors:** Josephine Sikulu, Ralf Kubini, Muath Turkman, Moritz Benjamin Immohr, Michael Becker, Rashad Zayat, Ali Aljalloud

**Affiliations:** ^1^ Department of Cardiology Luisenhospital Aachen Germany; ^2^ Department of Cardiology Rhein‐Maas Hospital, Nephrology and Internal Intensive Care Würselen Germany; ^3^ Department of Thoracic and Cardiovascular Surgery, West German Heart and Vascular Center, University Hospital Essen University of Duisburg‐Essen Essen Germany; ^4^ Department of Cardiac Surgery RWTH University Hospital Aachen Aachen Germany

**Keywords:** acetylcholine provocation, calcium‐channel blocker, coronary spasm, INOCA, women

## Abstract

**Background:**

Coronary vasomotor disorders are frequently underdiagnosed in women with angina and non‐obstructive coronary arteries. We investigated clinical presentation, diagnostic delay, spasm subtype, and patient‐reported treatment response in women with acetylcholine‐confirmed coronary spasm.

**Methods:**

This single‐center observational study included women with a positive intracoronary acetylcholine provocation test who completed a locally developed questionnaire on symptoms, triggers, comorbidities, medication tolerance, and treatment response. Epicardial spasm was defined as ≥ 90% epicardial diameter reduction with symptom reproduction and ischemic ECG changes; microvascular spasm as symptoms and ischemic ECG changes with < 90% epicardial vasoconstriction. Diltiazem was initiated as first‐line therapy, with amlodipine as an alternative. Regression analyses exploring therapeutic failure were considered exploratory because of the low event count.

**Results:**

A total of 194 women were included (median age 60 years [IQR 52–67]). Chest pain/tightness was reported by 163/190 (85.8%), dyspnea by 70/135 (51.9%), and palpitations by 74/136 (54.1%). Epicardial/microvascular spasm was present in 110/187 (58.8%) and 77/187 (41.2%). Median symptom‐to‐diagnosis time was 24 months [IQR 8–90]. Diltiazem was prescribed in 162/194 (83.5%). Among respondents with follow‐up data, symptom frequency decreased in 103/128 (80.5%), episode duration decreased in 91/118 (77.1%), and pain intensity decreased/resolved in 92/115 (80.0%). Therapeutic failure occurred in 8/106 (7.5%). Residual symptom burden remained common, with CCS class > II at follow‐up in 65/124 (52.4%) and persistent complaints in 53/107 (49.5%).

**Conclusions:**

In women with acetylcholine‐confirmed coronary spasm, symptom burden and diagnostic delay were substantial. Calcium‐channel blocker therapy was associated with patient‐reported symptom improvement among respondents with available follow‐up data.

## Graphical abstract

In women with acetylcholine‐confirmed coronary spasm, symptom burden and diagnostic delay were substantial. Calcium‐channel blocker therapy was associated with patient‐reported symptom improvement among respondents with available follow‐up data, although residual symptoms remained common.

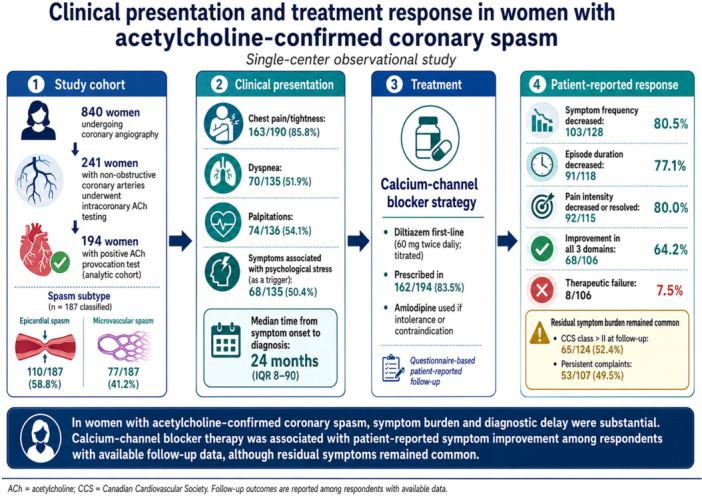

## 1 Introduction

Coronary spasm and coronary vasomotor disorders are important mechanisms of angina in patients without obstructive coronary artery disease (INOCA). In women undergoing coronary angiography for suspected ischemia, normal or non‐obstructive coronary arteries are common, yet symptom burden and recurrent healthcare contacts remain substantial [1, 2].

Intracoronary acetylcholine (ACh) provocation testing can unmask epicardial and microvascular spasm and has been shown to be feasible and safe in experienced centers [3]. Coronary vasomotor dysfunction in INOCA appears to be frequently dominated by vasospasm [4].

Sex‐specific patterns have been reported in coronary vasomotor disorders: in mixed cohorts undergoing ACh testing, women more often exhibit microvascular spasm, whereas men more often show epicardial spasm; long‐term outcomes appear broadly comparable, although prognostic implications of a positive ACh test may differ by sex [5, 6, 7, 8].

The international initiative *Coronary Vasomotor Disorders International Study Group* (COVADIS) has proposed standardized diagnostic criteria for VSA to facilitate clinical implementation and research comparability [9]. However, real‐world management in women, including diagnostic delay, symptom triggers, and treatment response to calcium‐channel blockers, remains incompletely described.

We therefore conducted a single‐center observational study of women with ACh‐confirmed coronary spasm, focusing on clinical presentation, diagnostic delay, and patient‐reported response to calcium‐channel blocker therapy.

## 2 Methods

### 2.1 Study Design and Population

This retrospective observational study was conducted from 01.09.2019 to 01.04.2021 at the Women's Heart Center of the Rhein‐Maas Hospital. Consecutive 840 women undergoing coronary angiography for suspected coronary artery disease based on angina, dyspnea and positive noninvasive stress test were eligible for inclusion.

During the study period, 840 women underwent coronary angiography. Obstructive coronary artery disease was present in 599 women (71.3%) and these patients were not eligible for ACh testing. The remaining 241 women (28.7%) had non‐obstructive coronary arteries and underwent intracoronary ACh provocation testing during the same session. A positive provocation test was observed in 194/241 women (80.5%) and these patients constitute the present analytic cohort. These figures describe the diagnostic flow of the study population and should not be interpreted as population‐level prevalence estimates.

All participants provided written informed consent for coronary angiography, ACh testing, and pseudonymized data analysis in compliance with German data protection legislation.

### 2.2 Acetylcholine Provocation Protocol and Definitions

ACh was administered manually over 1 min into the left coronary artery via an interventional catheter (EBU/JL) in incremental doses of 2 μg, 20 μg, 100 μg, and 200 μg, with angiography after each dose. Heart rate, blood pressure, and 12‐lead ECG were monitored continuously. Intracoronary nitroglycerin was administered to relieve provoked spasm.

Test interpretation was based on reproduction of symptoms (e.g., chest pain or dyspnea), ischemic ECG changes (transient ST‐segment depression or elevation ≥ 0.1 mV in at least two contiguous leads), and angiographic vasoconstriction. Epicardial spasm was defined as ≥ 90% diameter reduction relative to baseline with symptoms and ischemic ECG changes, consistent with prior invasive ACh testing definitions [3, 9]. Microvascular spasm was defined as symptoms and ischemic ECG changes with < 90% epicardial vasoconstriction [3, 9]. No ≥ 75% angiographic threshold was used for epicardial spasm classification in the present analysis. ECG changes and angiographic findings were assessed by the interventional operator during the procedure; independent core‐laboratory ECG or quantitative coronary angiographic adjudication was not performed.

Patients with spontaneous or catheter‐induced spasm, acute coronary syndrome, myocarditis, marked left ventricular systolic dysfunction, or haemodynamic instability were excluded from provocation testing.

### 2.3 Treatment and Follow‐Up

Patients with a positive test result were started on diltiazem 60 mg twice daily as first‐line therapy, with dose titration according to symptom control and tolerance. Amlodipine was used in cases of intolerance or contraindication to diltiazem.

Treatment response was assessed by a locally developed 14‐item questionnaire addressing symptom characteristics, triggers, comorbidities, medication tolerance, and patient‐reported changes in symptom frequency, episode duration, and pain intensity (visual analogue scale, VAS 0–10). The questionnaire was not formally validated and is provided as Supporting Information S1. Baseline VAS was obtained from the clinical questionnaire and may include retrospective recall in patients completing follow‐up questionnaires; therefore, recall bias cannot be excluded. Follow‐up timing was not standardized, and not all participants provided responses to all items; denominators are therefore reported for each variable.

### 2.4 Outcomes and Statistical Analysis

The primary descriptive outcomes were symptom patterns, diagnostic delay, spasm subtype, and patient‐reported treatment response. Therapeutic failure was defined a priori as no improvement in all three symptom domains (frequency, duration, and pain intensity). This stringent definition was chosen to identify patients with no patient‐reported benefit across all measured domains, but it may underestimate clinically relevant residual symptom burden; therefore, residual complaints, CCS class > II, and domain‐specific responses are reported separately.

Statistical analyses were performed using SPSS Statistics version 28.0.1.1 (IBM, Armonk, NY, USA). Continuous variables are presented as median (interquartile range), and categorical variables as *n* (%). Because questionnaire completion was incomplete, analyses were performed as complete‐case for each item (variable denominators). Univariate logistic regression was used to explore factors associated with therapeutic failure, and a limited multivariable model was fitted given the low event count. These models were considered exploratory and underpowered; causal or predictive interpretation was avoided. Two‐sided *p*‐values < 0.05 were considered statistically significant. Given the small number of therapeutic failure events, we performed an additional sensitivity analysis using Firth's penalized logistic regression to reduce small‐sample bias [10, 11].

## 3 Results

**FIGURE 1:**
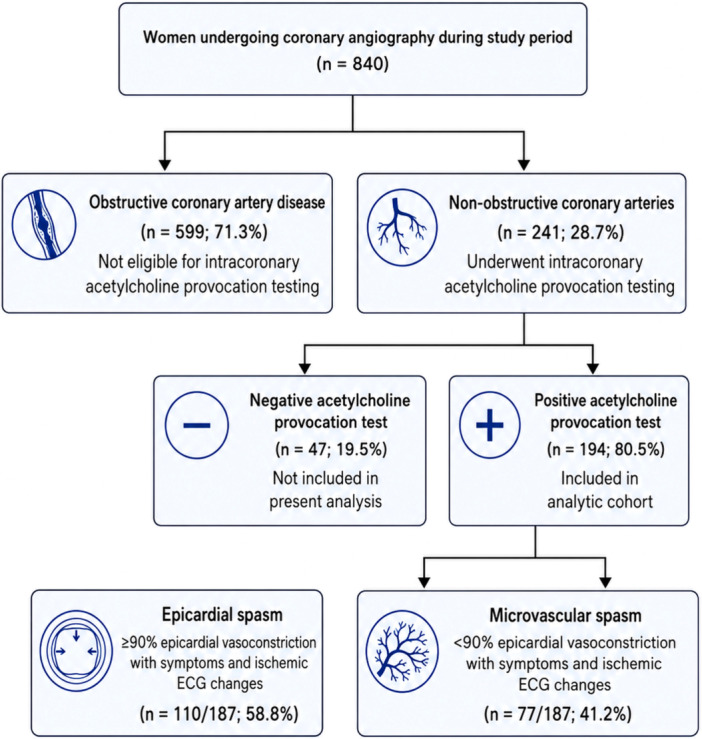
Study flow chart. Flow of women undergoing coronary angiography during the study period. Of 840 women, 599 (71.3%) had obstructive coronary artery disease and were ineligible for acetylcholine provocation testing. The remaining 241 women (28.7%) with non‐obstructive coronary arteries underwent testing; 194 (80.5%) had a positive test and formed the analytic cohort. Among 187 patients with available angiographic classification, epicardial spasm was present in 110 (58.8%) and microvascular spasm in 77 (41.2%). Percentages for obstructive versus non‐obstructive disease are based on all 840 women; acetylcholine test results on the 241 women with non‐obstructive arteries; and spasm subtype on 187 classified patients.

Within the study period, 840 women underwent coronary angiography and 194 women with a positive ACh provocation test were included (median age 60 years [IQR 52–67]; Table 1; Figure 1). Chest pain or tightness was the most common presenting symptom (163/190, 85.8%), followed by palpitations (74/136, 54.1%) and dyspnea (70/135, 51.9%) (Table 1). Symptoms were reported in association with psychological stress by 68/135 (50.4%) (Table 1). Stress was interpreted as a potential trigger of vasomotor instability and not as evidence of psychogenic symptoms.

**TABLE 1:** Baseline characteristics and symptom profile.

Variable	*n*	Value
Age, years	194	60.0 (52.0–67.0)
Body weight, kg	194	73.0 (64.0–84.0)
Body mass index, kg/m^2^	194	25.7 (22.9–29.4)
Chest pain/tightness	190	163 (85.8)
Dyspnea	135	70 (51.9)
Palpitations/tachycardia	136	74 (54.1)
Symptoms associated with psychological stress	135	68 (50.4)
Episodes ≥ 15 min	130	56 (43.1)
Episodes at least daily	130	55 (42.3)
Pain intensity at presentation (VAS 0–10)	123	7.0 (2.0–8.0)
Time since first symptom onset, months	121	24.0 (10.5–72.0)
Time until first presentation, months	96	6.0 (3.0–12.0)
Time until diagnosis, months	101	24.0 (8.0–90.0)
Previous incorrect diagnoses, *n*	102	2.0 (0.0–8.5)
Previously misdiagnosed as mental health issue	56	31 (55.4)
Arterial hypertension	136	43 (31.6)
Diabetes mellitus	136	7 (5.1)
Concomitant psychiatric illness	135	33 (24.4)
Dyslipidemia	135	15 (11.1)
Smoking	135	65 (48.1)
Alcohol > 1 × /week	136	33 (24.3)
Family history of coronary artery disease	135	72 (53.3)
Left ventricular ejection fraction, %	162	59.0 (56.0–62.0)
Diastolic dysfunction	159	14 (8.8)
Interventricular septal end‐diastole, mm	155	9.0 (8.0–10.0)

*Note:* Values are presented as median (interquartile range) or *n* (%), as appropriate. Percentages are calculated using the available denominator for each variable.

Abbreviations: BMI, body mass index; IQR, interquartile range; VAS, visual analogue scale.

During ACh testing, angina was provoked in 167/188 (88.8%) and ischemic ECG changes occurred in 130/189 (68.8%) (Table 2). Angiographic vasoconstriction ≥ 90%, consistent with epicardial spasm, was observed in 110/187 (58.8%). Microvascular spasm, defined as symptoms and ischemic ECG changes with < 90% epicardial vasoconstriction, was observed in 77/187 (41.2%) (Table 2). Symptoms resolved after intracoronary nitroglycerin in 170/171 (99.4%) (Table 2).

**TABLE 2:** Acetylcholine provocation testing findings.

Finding	*n*	Value
Ischemic ECG changes during ACh test	189	130 (68.8)
Angina pectoris during ACh test	188	167 (88.8)
Shortness of breath during ACh test	183	4 (2.2)
Vasoconstriction ≥ 90%	187	110 (58.8)
Recovery after intracoronary nitroglycerin	171	170 (99.4)

*Note:* Values are presented as *n* (%). Percentages are calculated using the available denominator for each finding.

Abbreviations: ACh, acetylcholine; ECG, electrocardiogram.

**FIGURE 2:**
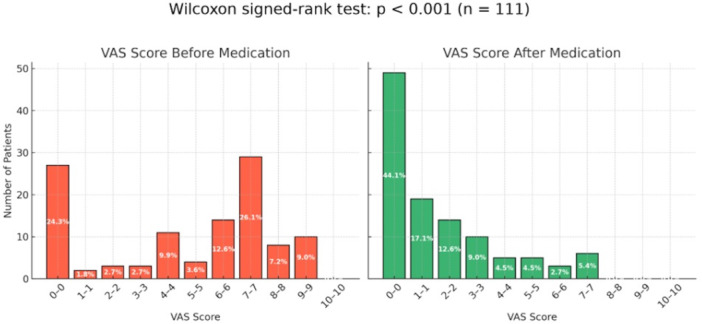
Chest pain intensity before and after medication. Distribution of visual analogue scale (VAS, 0–10) scores at baseline and follow‐up among patients with paired responses (*n* = 111). Wilcoxon signed‐rank test *p* < 0.001.

**FIGURE 3:**
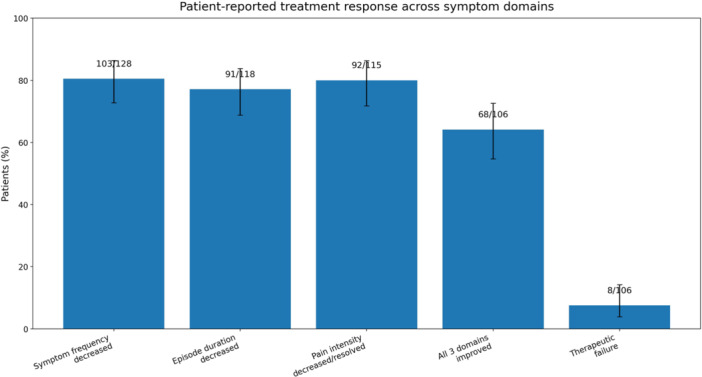
Patient‐reported treatment response across symptom domains. Bar chart summarizing patient‐reported improvement in symptom frequency, episode duration, and pain intensity (VAS), as well as improvement in all three domains and therapeutic failure among respondents with available data (n as indicated above bars). Error bars indicate 95% Wilson confidence intervals.

Diltiazem was prescribed in 162/194 (83.5%) (Table 3). Among respondents with available follow‐up data, symptom frequency decreased in 103/128 (80.5%), episode duration in 91/118 (77.1%), and pain intensity decreased or resolved in 92/115 (80.0%); median follow‐up VAS was 2 (IQR 0–4) (Table 3; Figure 2). Improvement in all three domains was observed in 68/106 (64.2%), whereas therapeutic failure occurred in 8/106 (7.5%) (Table 3; Figure 3). However, residual symptom burden remained common: 65/124 (52.4%) had CCS class > II at follow‐up and 53/107 (49.5%) reported persistent complaints.

**TABLE 3:** Pharmacotherapy and patient‐reported outcomes at follow‐up.

Outcome	*n*	Value
Diltiazem prescribed	194	162 (83.5)
Still on diltiazem at late follow‐up	107	65 (60.7)
Good tolerance of pharmacotherapy	129	101 (78.3)
Reduction of symptom frequency	128	103 (80.5)
Reduction of episode duration	118	91 (77.1)
Pain intensity decreased or resolved	115	92 (80.0)
Pain (VAS) at follow‐up	119	2.0 (0.0–4.0)
Improvement in frequency, duration, and VAS	106	68 (64.2)
Therapeutic failure	106	8 (7.5)
CCS classification > Class II at follow‐up	124	65 (52.4)
Complaints at late follow‐up	107	53 (49.5)

*Note:* Values are presented as median (interquartile range) or *n* (%), as appropriate. Percentages are calculated using the available denominator for each outcome.

Abbreviations: CCS, Canadian Cardiovascular Society; IQR, interquartile range; VAS, visual analogue scale.

Follow‐up timing was not uniform. Exact questionnaire completion dates were not consistently available, so the median interval from diagnosis or treatment initiation to follow‐up questionnaire completion could not be calculated. Exact medication exposure duration was unavailable for all respondents; therefore, treatment‐response results are reported descriptively and restricted to participants with available data.

The distribution of episode duration change did not differ by baseline episode duration category (*p* = 0.83; Supporting Information S1: Figure 1).

**FIGURE 4:**
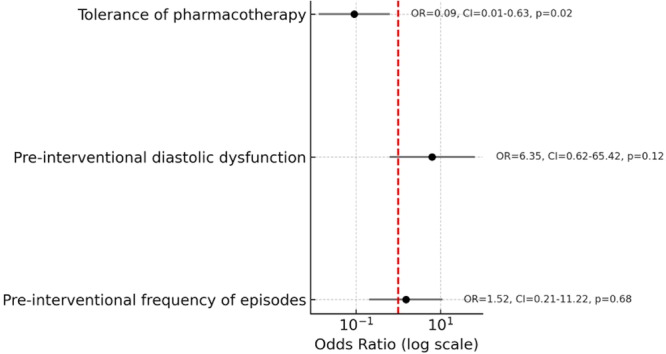
Exploratory factors associated with therapeutic failure (forest plot). Forest plot showing odds ratios (log scale) from multivariable logistic regression for therapeutic failure. Error bars indicate 95% confidence intervals.

In exploratory regression analyses (eight therapeutic failure events), diastolic dysfunction was associated with higher odds of therapeutic failure in univariate analysis (OR 7.60, 95% CI 1.11–52.02; *p* = 0.04), while good tolerance of pharmacotherapy was associated with lower odds (OR 0.11, 95% CI 0.02–0.52; *p* < 0.01). In multivariable analysis, tolerance remained associated with lower odds of failure (OR 0.09; *p* = 0.02), whereas diastolic dysfunction was not statistically significant (OR 6.35; *p* = 0.12) (Table 4; Figure 4). Because only eight therapeutic failure events occurred and confidence intervals were wide, these estimates, particularly for diastolic dysfunction, are imprecise and should be interpreted only as hypothesis‐generating.

**TABLE 4:** Exploratory factors associated with therapeutic failure.

Variable	Univariate OR (95% CI)	*p*	Multivariable OR	*p*
Episodes at least daily	4.05 (0.78–21.12)	0.10	1.52	0.68
Diastolic dysfunction	7.60 (1.11–52.02)	0.04	6.35	0.12
Good tolerance of pharmacotherapy	0.11 (0.02–0.52)	< 0.01	0.09	0.02

*Note:* Odds ratios are derived from logistic regression. Analyses were exploratory because of the low number of therapeutic failure events.

Abbreviations: CI, confidence interval; OR, odds ratio.

In complete‐case sensitivity analyses based on paired symptom‐domain responses (*n* = 103), therapeutic failure occurred in 13/103 (12.6%). By spasm subtype, therapeutic failure occurred in 5/58 patients with epicardial spasm (8.6%) and 8/45 patients with microvascular spasm (17.8%; *p* = 0.233; Supporting Information S1: Table 1). In a Firth penalized logistic regression model (Supporting Information S1: Figure 2), good tolerance of pharmacotherapy was associated with lower odds of therapeutic failure (OR 0.22, 95% CI 0.06–0.74; *p* = 0.015), whereas diastolic dysfunction and baseline episode frequency were not statistically significant (Supporting Information S1: Table 1). In addition, follow‐up pain intensity (VAS) was lower among patients reporting good tolerance (median 2 [IQR 0–3] vs. 4 [IQR 1–6]; *p* = 0.010; Supporting Information S1: Figure 3).

## 4 Discussion

In this single‐center observational cohort of women with ACh‐confirmed coronary spasm, symptom burden was high and the time to diagnosis was long. Beyond chest pain, dyspnea and palpitations were frequent, and psychological stress was commonly reported as a trigger.

These findings align with prior work showing that women with angina and non‐obstructive coronary arteries often have persistent symptoms and recurrent healthcare contacts, frequently driven by microvascular dysfunction and vasomotor abnormalities [1, 12, 13, 14, 15, 16]. In sex‐comparative ACh cohorts, women show a higher prevalence of microvascular spasm and a lower prevalence of epicardial spasm, which may contribute to symptom heterogeneity and diagnostic uncertainty [6, 7, 8]. Mechanistically, coronary artery spasm reflects a spectrum of endothelial dysfunction and vascular smooth muscle hyperreactivity, which may be exacerbated by autonomic and psychosocial stressors; genetic variants that reduce endothelial nitric oxide synthase expression (e.g., the eNOS T‐786C promoter variant) have been associated with coronary spasm, supporting a role of impaired NO bioavailability [17],

ACh provocation testing provides a physiologic diagnosis and can differentiate epicardial from microvascular spasm when angiographic constriction is limited despite ischemic ECG changes and symptom reproduction [2, 3, 14]. In the present analysis, epicardial spasm was classified only when ≥ 90% epicardial diameter reduction occurred together with symptoms and ischemic ECG changes. Harmonized definitions remain important for comparability across studies, particularly when mixed epicardial and microvascular spasm cohorts are analyzed [9].

Calcium‐channel blockers are recommended as first‐line therapy for vasospastic angina and were generally well tolerated in our cohort, with most respondents reporting improvement across multiple symptom domains [12, 18, 19, 20]. However, because follow‐up was questionnaire‐based, timing was not standardized, medication exposure was incompletely quantified, and no untreated control group was available, the observed improvements should not be interpreted as proof of treatment efficacy. Rather, these data describe real‐world patient‐reported outcomes after a treatment strategy centered on calcium‐channel blocker therapy. Given that coronary spasm can occasionally present with malignant arrhythmias or catastrophic clinical courses, early recognition and targeted therapy are clinically relevant [21]. Our data support structured INOCA pathways that include coronary function testing in symptomatic women after non‐obstructive angiography [2, 9].

In exploratory regression analyses, diastolic dysfunction and poorer medication tolerance were associated with therapeutic failure; however, only eight failure events occurred and confidence intervals were wide, particularly for diastolic dysfunction. Accordingly, these associations should be considered hypothesis‐generating, and future studies should pre‐specify responder definitions and use penalized or Firth logistic regression when event counts are low.

## 5 Limitations

This study has limitations inherent to its retrospective design and questionnaire‐based follow‐up. Not all participants completed all questionnaire items, resulting in variable denominators and potential response bias; percentages are therefore reported relative to available responses and treatment‐response findings apply only to respondents with available follow‐up data. The 14‐item questionnaire was locally developed and not formally validated, and baseline symptom intensity may have been subject to retrospective recall bias. Follow‐up timing, exact questionnaire completion dates, dose titration, medication changes, adherence, and exact treatment exposure duration could not be standardized. Angiographic vasoconstriction was assessed in routine clinical practice without independent core‐laboratory quantitative coronary angiography, and visual estimation of percent vasoconstriction may affect classification of epicardial versus microvascular spasm. ECG changes during ACh testing were assessed by the operator and were not independently adjudicated. The cohort included only women with a positive ACh test at a referral center and therefore should not be used to estimate population‐level spasm prevalence. There was no control group, and the small number of therapeutic failure events limits the robustness of multivariable modeling.

## 6 Conclusion

Among women with acetylcholine‐confirmed coronary spasm, symptom burden and diagnostic delay are substantial. Calcium‐channel blocker therapy was associated with patient‐reported symptom improvement among respondents with available follow‐up data, but residual symptoms remained common. Prospective studies with standardized follow‐up, harmonized spasm definitions, validated patient‐reported outcomes, and quantitative assessment of medication exposure are warranted.

## Author Contributions

Josephine Sikulu and Ali Aljalloud conceived and designed the study and developed the study protocol. Josephine Sikulu was responsible for data collection, data interpretation, and drafting the manuscript. Muath Turkman contributed to patient follow‐up and data collection. Moritz Benjamin Immohr and Rashad Zayat performed the statistical analyses and contributed to data interpretation. Ralf Kubini and Michael Becker contributed to the practical conduct of the study and interpretation of the findings. Ali Aljalloud supervised the study, contributed to the practical conduct of the study, participated in data interpretation and statistical analysis, and critically revised the manuscript. All authors reviewed and approved the final manuscript.

## Funding

The authors have nothing to report.

## Ethics Statement

This study was approved by the Ethics Committee of the Medical Faculty of RWTH Aachen University (MTI 2, Wendlingweg 2, 52074 Aachen, Germany; approval number: EK250/21).

## Conflicts of Interest

The authors declare no conflicts of interest.

## Supporting information


Supporting File

## Data Availability

The data that support the findings of this study are available on request from the corresponding author. The data are not publicly available due to privacy or ethical restrictions.
