# Supplementary material for: Clinical Presentation and Treatment Response in Women With Acetylcholine‐Confirmed Coronary Spasm: A Single‐Center Observational Study
Source: Clin Cardiol. 2026 Jul 21;49(7):e70424. doi: 10.1002/clc.70424 (PMC13386483; doi:10.1002/clc.70424)
Supplement: Supplementary file 1 — Supporting File [file CLC-49-e70424-s001.docx]

# Supplementary material

**Table S1. Subgroup analysis of therapeutic failure in the complete-case cohort.**

| Variable | Therapeutic failure | No therapeutic failure | p value |
| --- | --- | --- | --- |
| Good tolerance of pharmacotherapy | 6/13 (46.2%) | 75/90 (83.3%) | 0.006 |
| Diastolic dysfunction (≥ grade I) | 2/13 (15.4%) | 5/90 (5.6%) | 0.214 |
| ECG changes during ACh test | 9/13 (69.2%) | 63/90 (70.0%) | 1 |
| Epicardial spasm / vasoconstriction ≥90% during ACh test | 5/13 (38.5%) | 53/90 (58.9%) | 0.233 |
| Age, years | 65.0 (59.0–71.0) | 60.0 (55.0–66.0) | 0.131 |
| BMI, kg/m² | 24.2 (23.8–27.4) | 25.2 (22.5–29.3) | 0.575 |
| Episode frequency before treatment (ordinal 1–3) | 3.0 (3.0–3.0) | 2.0 (2.0–3.0) | 0.051 |
| VAS before medication (0–10) | 5.0 (0.0–8.0) | 7.0 (3.0–8.0) | 0.118 |

Values are n/N (%) or median (IQR). P values from Fisher’s exact test (binary variables) or Mann–Whitney U test (continuous/ordinal variables).

**Supplementary Figure S1. Change in episode duration after treatment by baseline episode duration.**


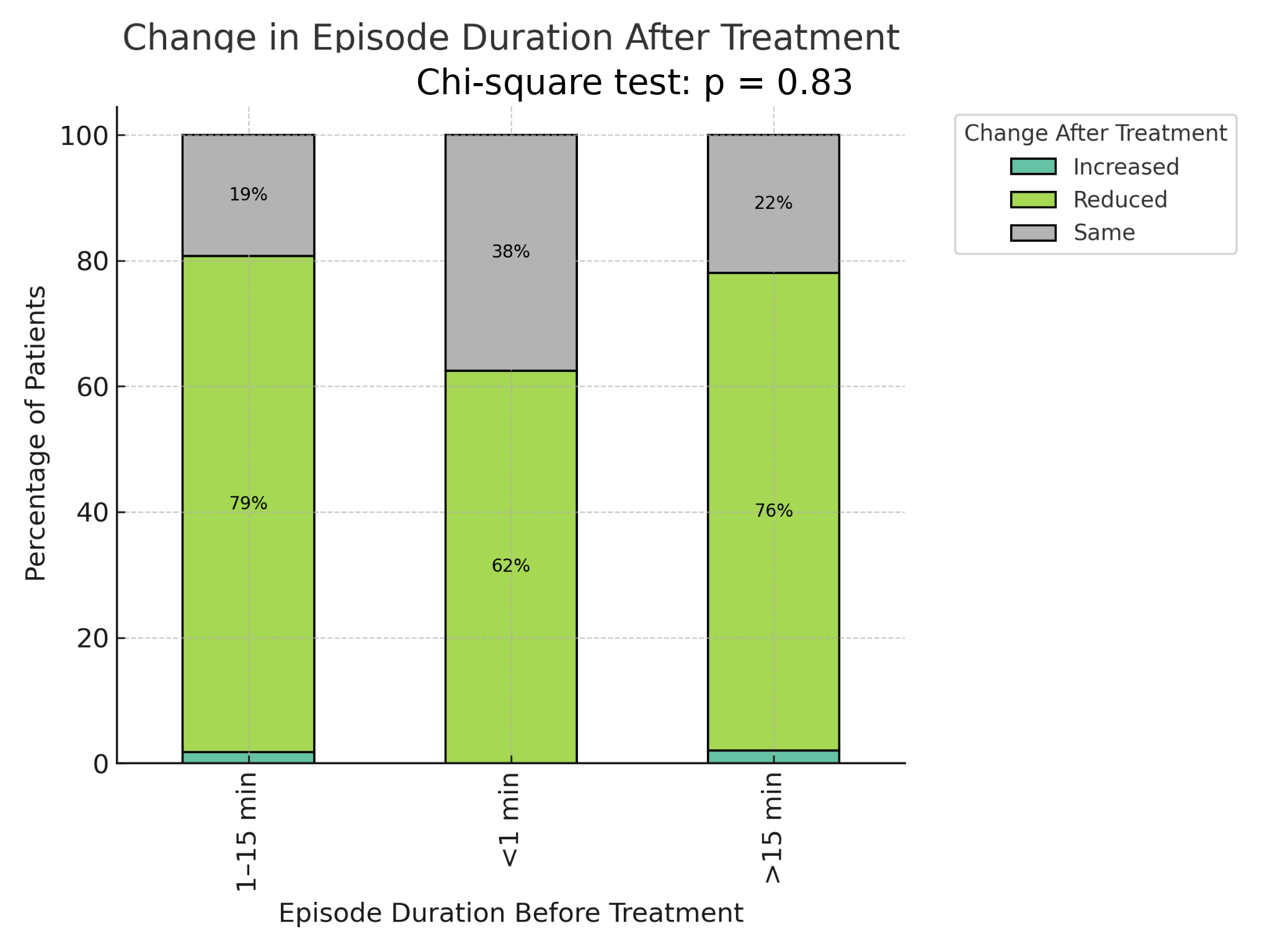


Stacked bar chart showing the proportion of patients reporting increased, reduced, or unchanged episode duration at follow-up stratified by baseline episode duration category. Chi-square test p = 0.83.

**Figure S2. Firth penalized logistic regression for therapeutic failure.**


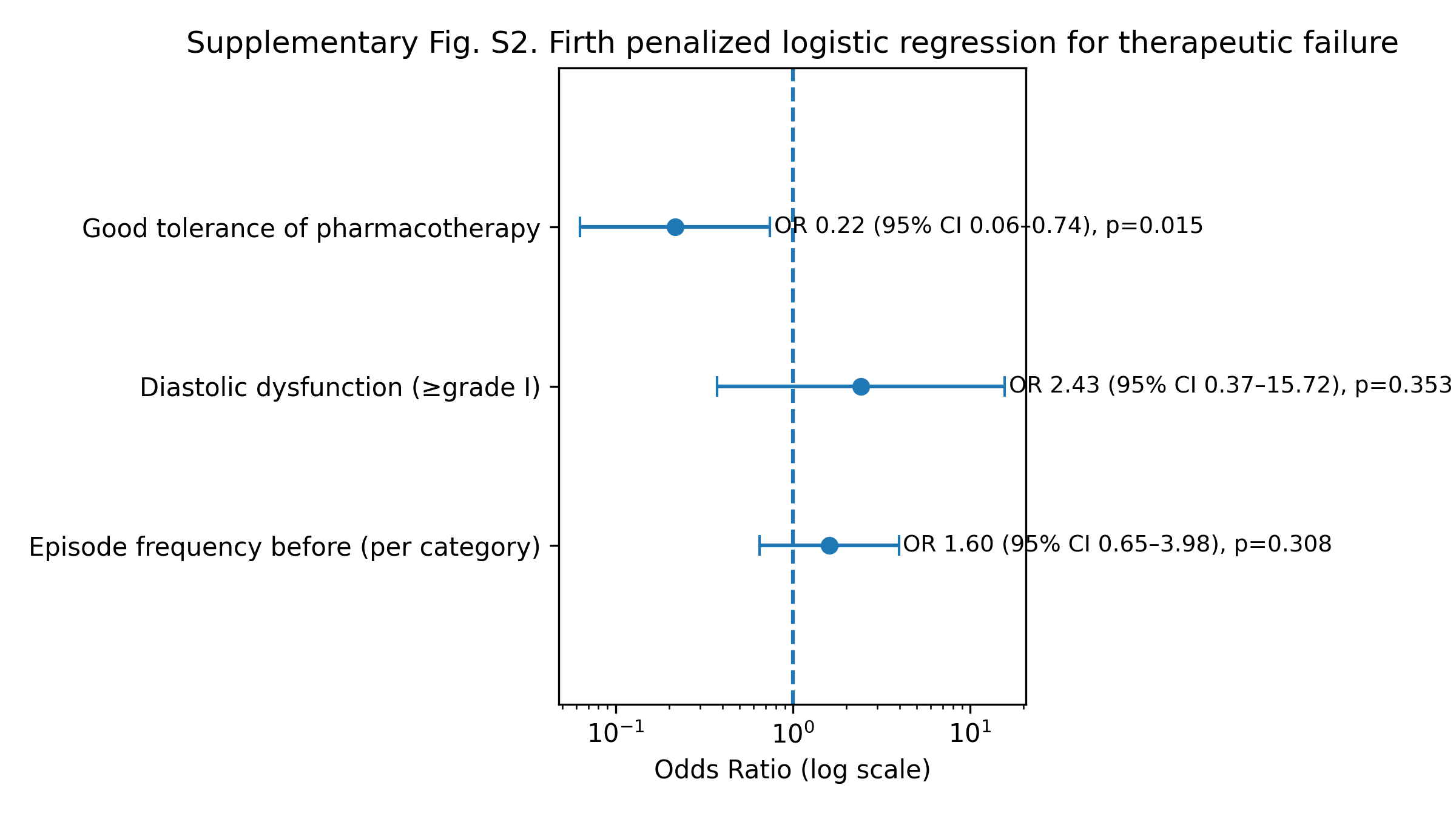


Forest plot showing odds ratios (log scale) and 95% confidence intervals from Firth penalized logistic regression in the complete-case cohort.

**Figure S3. Follow-up pain intensity by tolerance of pharmacotherapy.**


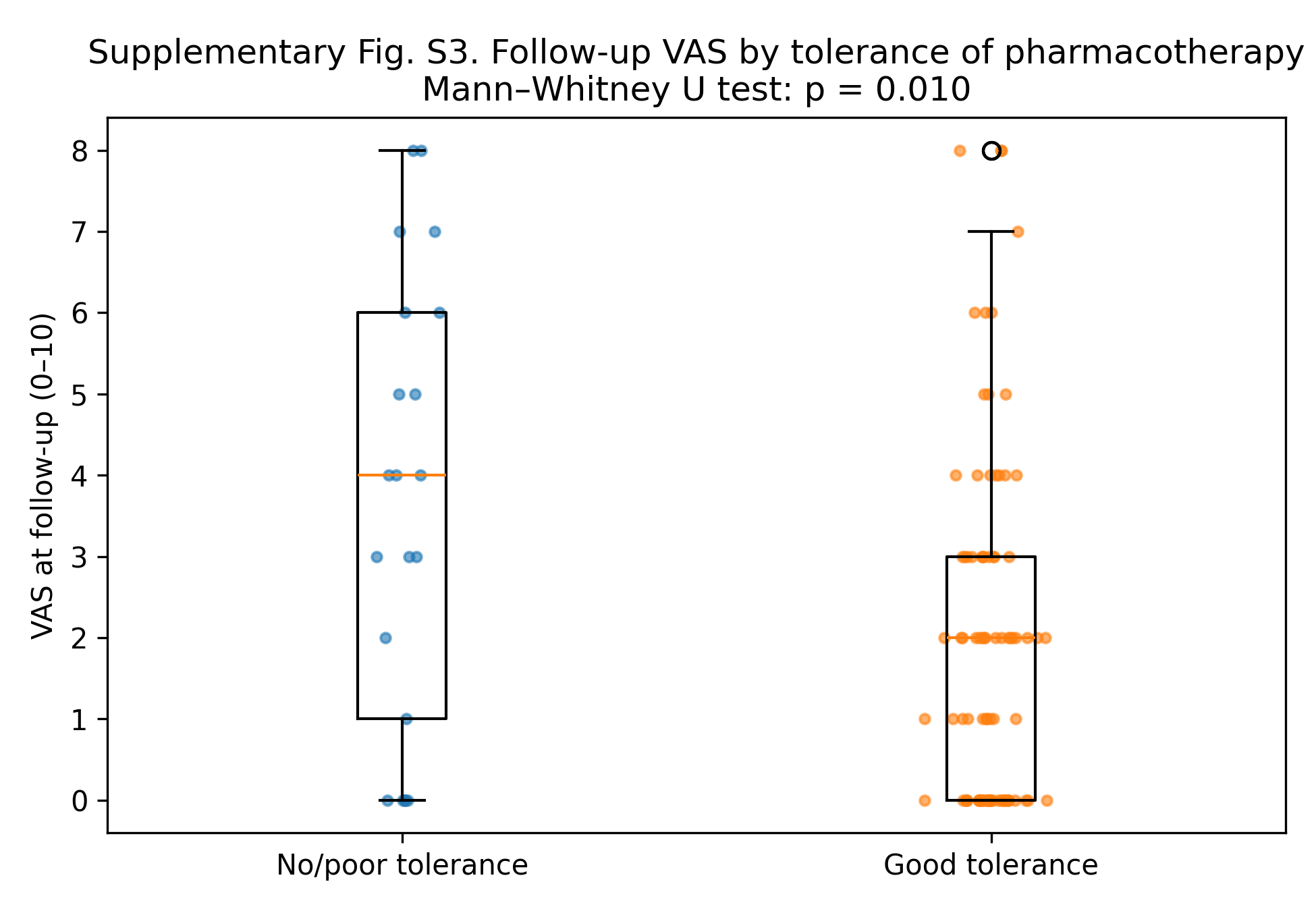


Boxplots show VAS at follow-up stratified by patient-reported tolerance of pharmacotherapy. Points represent individual patients; p value from Mann–Whitney U test.
